# Steam Explosion Pretreatment of Polysaccharide from *Hypsizygus marmoreus*: Structure and Antioxidant Activity

**DOI:** 10.3390/foods13132086

**Published:** 2024-07-01

**Authors:** Zirong Huang, Yueyue Qiang, Shiyu Zhang, Yujia Ou, Zebin Guo, Baodong Zheng

**Affiliations:** 1 College of Food Science, Fujian Agriculture and Forestry University, Fuzhou 350002, China; hzrfst@163.com (Z.H.); qyydsw@163.com (Y.Q.); 18120909018@163.com (S.Z.); oyjfst@163.com (Y.O.); gzb8607@163.com (Z.G.); 2 Integrated Scientific Research Base of Edible Fungi Processing and Comprehensive Utilization Technology, Ministry of Agriculture and Rural Affairs, Fuzhou 350002, China; 3 Key Laboratory of Subtropical Characteristic Fruits, Vegetables and Edible Fungi Processing (Co-Construction by Ministry and Province), Ministry of Agriculture and Rural Affairs, Fuzhou 350002, China; 4 China-Ireland International Cooperation Centre for Food Material Science and Structure Design, Fujian Agriculture and Forestry University, Fuzhou 350002, China

**Keywords:** *Hypsizygus marmoreus*, polysaccharide, structural characterization, antioxidant

## Abstract

This paper investigated the effects of steam explosion (SE) pretreatment on the structural characteristics and antioxidant activity of *Hypsizygus marmoreus* polysaccharides (HPS). *Hypsizygus marmoreus* samples were pretreated at different SE temperatures (120–200 °C) and polysaccharides were extracted using the water extraction and alcohol precipitation method. The results showed that SE pretreatment improved the extraction rate of HPS. Under the conditions of SE treatment time of 60 s and temperature of 160 °C, the extraction rate of HPS was the highest (8.78 ± 0.24%). After SE pretreatment, the structural changes of HPS tended to enhance the antioxidant activity, which showed that the content of Gal and Man in the monosaccharide composition increased and the molecular weight decreased. When testing antioxidant activity in vitro, the ability of SE-pretreated HPS to scavenge DPPH radicals, hydroxyl radicals, and superoxide anion radicals was better than that of HPS without SE pretreatment. Our findings shed light on SE pretreatment as an efficient method for extracting active polysaccharides, providing a new way to improve their extraction rate and biological activity.

## 1. Introduction

*Hypsizygus marmoreus* (Peck) H. E. Bigelow (*H. marmoreus*) is a basidiomycete of high edibility and medicinal value that is widely distributed in Asia, mainly in Japan, Korea, and China, especially in the Shanxi, Shandong, and Fujian Provinces in China [1,2]. Dried *H. marmoreus* contains 21.85% protein, 2.74% crude fiber, 3.64% crude fat, 7.64% ash, and 4.8% polysaccharides [3]. *H. marmoreus* polysaccharides (HPS) are an essential source of active polysaccharides, mainly consisting of rhamnose (Rha), mannose (Man), galactose (Gal), and glucose (Glc) [4]. They also contain abundant antioxidant groups such as carboxyl, carbonyl, and ester [3], which have anti-inflammatory activity [5], anti-tumor activity [6], and antioxidant activity [4]. Among them, Liu et al. [4,7] extracted three polysaccharides (EEPS, AEPS, EPS) from *H. marmoreus* by three different extraction methods and fed them to mice. It was found that the activities of superoxide dismutase (SOD), glutathione peroxidase (GSH–Px), catalase (CAT), and total antioxidant capacity (T–AOC) in the renal tissue of mice were significantly increased, and the contents of lipid peroxidase (LPO) and malondialdehyde (MDA) were significantly decreased, indicating that polysaccharides from *H. marmoreus* have significant antioxidant activities in vivo. Monosaccharide composition, functional groups, molecular weight, and surface structure of polysaccharides are essential factors that determine polysaccharide bioactivity [8]. Polysaccharides are rich in Gal and Man and have been shown to have high antioxidant activity [9,10]. In addition, the microstructures of polysaccharides affect their biological activity. The more porous and expansive the surface structures of polysaccharides, the stronger their biological activity is. This is because of the increase in their specific surface area, which promotes the exposure of binding sites and functional groups [11]. Efficient extraction of bioactive polysaccharides is critical to exploring their application in functional foods.

Steam explosion (SE) is the steaming of raw materials at high pressure and temperature so that the steam is ultimately forced into the fabric. The high pressure is then released in milliseconds [12]. During the process, the water in the sample quickly swells and overflows, resulting in tremendous shear forces. This destroys the structure and chemical composition of the raw materials and improves the properties of the materials produced [13]. SE technology is widely used in food, medicine, bioenergy, and other industries to improve the yields of dietary fibers and polysaccharides [14]. Compared with other pretreatment methods, SE technology has the advantages of high efficiency, low cost, and good environmental sustainability [15]. Currently, SE is widely used for the modification of dietary fiber [12], proteins [16], cellulose [17], and polysaccharides [18]. Research has found that SE pretreatment of raw materials can improve their biological activity. Liang et al. compared the polysaccharides of Poria cocos extracted after SE pretreatment (SEPCP) and the polysaccharides of Poria cocos extracted without SE pretreatment (PCP) and found that SEPCP and PCP had the same monosaccharide composition. Still, the SEPCP had lower molecular weight and better cytophagy ability, indicating that within a certain range of molecular weight (1.2 × 10^4^–8.48 × 10^5^ Da), low molecular weight polysaccharides have strong biological activity [14]. Yi et al. [19] found that SE pretreatment can increase the content of glucuronic acid (GlcA), galactose (Gal), arabinose (Ara), and galacturonic acid (GalA), thereby enhancing antioxidant activity. Hu et al. [20] found that the scavenging rates of the DPPH radicals and hydroxyl radicals of wheat germ polysaccharides after SE pretreatment were higher than those without SE pretreatment. Chen et al. [21] found that after SE pretreatment, the molecular weight of the polysaccharides from *Polygonatum cyrtonema* decreased, Gal increased, and β-1,4-Galp, and β-1,4-Manp increased; hence, significantly increasing the scavenging activity of ABTS radicals. In summary, SE technology is an efficient and energy-saving modification method for increasing polysaccharide yield and enhancing the polysaccharide biological activity.

However, there are few studies on the extraction of HPS by SE pretreatment, and information about the relationship between the structural changes and functional activities of *H. marmoreus* after SE pretreatment is lacking. In general, the different temperatures during SE pretreatment depend on the pressure, which has a significant influence on the biological activity of SE-pretreated edible mushrooms. Consequently, this paper examined the effects of SE pretreatment at different temperatures (120–200 °C) on the structural and antioxidant activities of HPS, as well as investigating the correlations between these properties. The research results can influence the development of SE technology in the extraction and biological activity of edible fungi polysaccharides.

## 2. Materials and Methods

### 2.1. Materials and Chemicals

The *H. marmoreus* in this experiment was purchased from Fujian Shunchang Raoshi Baiyu Food Co., Ltd. (Nanping, China). The 1,1-diphenyl-2-picrylhydrazyl (DPPH) free radical scavenging detection kit, hydroxyl radical scavenging detection kit and superoxide anion free radical scavenging detection kit were purchased from Beijing Solarbio Science & Technology Co., Ltd. (Beijing, China). All other chemicals were of analytical grade.

### 2.2. Steam Explosion Pretreatment of H. marmoreus

We soaked the dried *H. marmoreus* samples in distilled water for 30 min before adding them to the SE reaction kettle (QB-200; Suzhou Tsing-Gentle Eco-technology Co., Suzhou, China). After the lid was closed, saturated steam was introduced into the reaction kettle to achieve the set pressure, and it was maintained for 60 s [22]. The obtained *H. marmoreus* samples were divided into the following six groups: control group (HPS-CK), 0.1 MPa (HPS-120 °C), 0.26 MPa (HPS-140 °C), 0.52 MPa (HPS-160 °C), 0.9 MPa (HPS-180 °C), and 1.45 MPa (HPS-200 °C). After the pressure maintenance, the pneumatic valve was opened to complete the SE. The wet material discharged from the chamber was the *H. marmoreus* after SE pretreatment. Finally, the *H. marmoreus* samples were dried in a drying oven (DS-30HL; Shanghai consistent constant temperature equipment center, Shanghai, China) at 55 °C for 24 h.

### 2.3. Extraction of HPS

The dried *H. marmoreus* samples were added to anhydrous ethanol at 90 °C to remove lipids by reflux and evaporated to dry at room temperature [23]. The supernatant was extracted with deionized water (1:20 *w*/*v*) at 90 °C for 2.5 h, centrifuged at 10,000× *g* for 15 min, and concentrated in a rotary evaporator. After that, pre-cooled anhydrous ethanol with triploid volume was added to the polysaccharide water extract and the mixture was refrigerated at 4 °C for 12 h, centrifuged to collect precipitation, and dissolved in water. Protein and pigment were removed and the product was dialyzed for 72 h and finally freeze-dried (CTFD-100S; Qingdao Yonghe Innovation Electronics Co., Ltd., Qingdao, China) to obtain HPS [24]. According to the above method, polysaccharides were extracted from six groups of *H. marmoreus* samples, and extraction was repeated three times per group. The quality of HPS was recorded as *A*_1_, and the rate of *H. marmoreus* extraction was recorded as *A*_2_.

(1) $$\mathrm{HPS}\;\mathrm{extraction}\;\mathrm{rate}\;(\%)=(A_{1}/A_{2})\times 100\%$$

### 2.4. Analysis of HPS Components

#### 2.4.1. Determination of Molecular Weight of HPS

The HPS sample was dissolved in 0.1 M NaNO_3_ and filtered through a 0.22 μm filter membrane into a 2 mL injection vial. The molecular weight distribution of the HPS was analyzed on two PL aqua gel-OH mixed 8 μm columns and an Agilent Infinity1260 instrument (Agilent Technology Co., Beijing, China) [25]. The molecular weight of the HPS was obtained after 30 min of determination [26]. 

#### 2.4.2. Determination of Monosaccharide Composition of HPS

**Figure 1:**
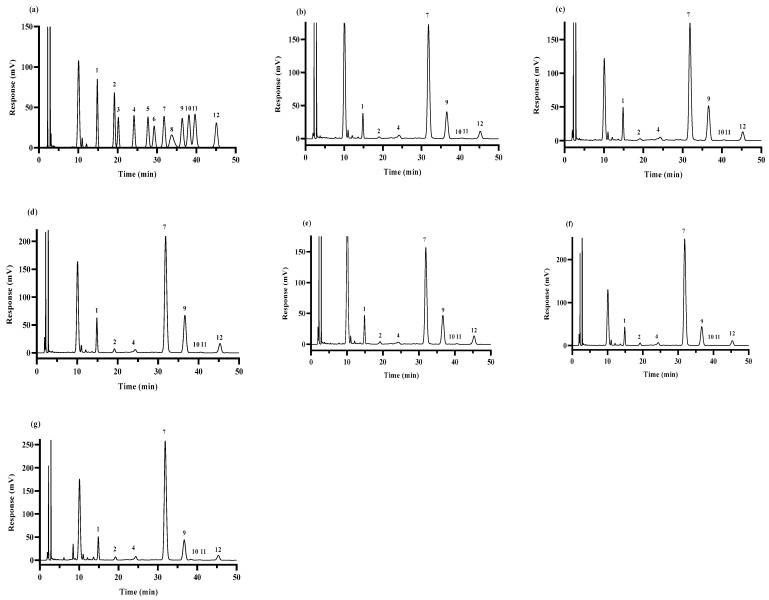
GC–MS chromatogram of standard monosaccharides (**a**); HPS-CK (**b**); HPS-120 °C (**c**); HPS-140 °C (**d**); HPS-160 °C (**e**); HPS-180 °C (**f**) and HPS-200 °C (**g**). Peaks: 1-Man; 2-Rib; 3-Rha; 4-GlcA; 5-GalA; 6-Nag; 7-Glc; 8-Gala; 9-Gal; 10-Xyl; 11-Ara; and 12-Fuc.

The monosaccharide standard is shown in Figure 1a. Standard products (mannose, ribose, rhamnose, glucuronic acid, galacturonic acid, N-acetyl-glucosamine, glucose, galactose, xylose, arabinose, and fucose) along with the polysaccharide samples were subjected to hydrolysis using trifluoroacetic acid. Subsequently, derivatization was carried out using propionate propylene glycol methyl ether, and the resulting derivatives were analyzed using high-performance liquid chromatography (HPLC) (Shimadzu LC-20AD, Kyoto, Japan). The detection conditions were a column temperature of 30 °C, flow rate of 1.0 mL/min, detection wavelength of 250 nm, the sample size of 20 μL, and a mobile phase of 0.05 M KH_2_PO_4_ (sodium hydroxide was used to adjust the pH to 6.7) [27].

#### 2.4.3. Determination of Total Sugar Content of HPS

We weighed Glc and dried it to constant weight, accurately prepared the standard solution of Glc of each concentration, added 2.5 mL of concentrated sulfuric acid and 0.5 mL of 6% phenol solution, heated it at 100 °C for 10 min, and then cooled the mixed solution. The absorbance value was determined at 490 nm by spectrophotometer (Shanghai Youke Instrument Co., Shanghai, China) [28]. The standard curve was obtained as follows: y=8.58x+0.05 (*R*^2^ = 0.98).

According to the standard curve, we weighed the *H. marmoreus* powder and repeated the above operation to calculate the HPS total sugar content.

#### 2.4.4. Determination of Protein Content of HPS

The HPS sample was diluted to a specific concentration by adding distilled water, of which 0.5 mL was absorbed, and 5 mL of 1 × G250 dyeing solution was added. The absorbance value was determined at 595 nm by a spectrophotometer [29]. The standard curve was obtained as follows: y=1.01x+0.54 (*R*^2^ = 0.98).

### 2.5. Structural Characteristics of HPS

#### 2.5.1. Infrared Spectroscopy (FT-IR) of HPS

The infrared spectrum of the HPS in the 4000–400 cm^−1^ region was obtained by an FT-IR spectrometer (Nicolet iS5 FT-IR, Thermo Scientific, New York, NY, USA) at room temperature (22 °C to 25 °C). Each sample was prepared by mixing HPS powder (1.0 mg) with KBr (20 mg). The scan time was 32 min, and the resolution was 4 cm^−1^ [30].

#### 2.5.2. Thermal Analyses of HPS

A TGA 8000 thermogravimetric analyzer (Perkinelmer Management Shanghai Co., Shanghai, China) was used for the thermogravimetric analysis (TGA) of the HPS. We placed the HPS in the crucible and heated it from 30 °C to 800 °C to observe its mass change. The carrier gas was N_2_, and the heating rate was 20 °C/min [31].

### 2.6. Determination of Antioxidant Activity of HPS In Vitro

#### 2.6.1. Determination of DPPH Radical Scavenging Rate by HPS

The HPS sample was prepared with distilled water into 3, 6, 9, 12, and 15 mg/mL sample solutions with five concentrations. According to the operating instructions of the kit, we added the reagent, and prepared the blank tube, the measuring tube, and the charge. The absorbance value was determined at 515 nm by allowing it to stand for 30 min away from light [32], denoted as *A*_1_, *A*_2_, and *A*_3_. Ascorbic acid was used as a positive control. The DPPH radical scavenging activity of the HPS was calculated according to the following equation:

(2) $$\mathrm{scavenging}\;\mathrm{percentage}\;(\%)=[A_{1}-(A_{2}-A_{3})/A_{1}]\times 100\%$$

#### 2.6.2. Determination of Hydroxyl Radical Scavenging Rate by HPS

The HPS sample was prepared with distilled water into 3, 6, 9, 12, and 15 mg/mL sample solutions with five concentrations. According to the operating instructions of the kit, we added the reagent, and prepared the blank tube, the measuring tube, and the charge. After reacting at 37 °C for 1 h, the absorbance value was determined at 536 nm [33], and denoted as *A*_1_, *A*_2_, and *A*_3_. Ascorbic acid was used as a positive control. The hydroxyl radical scavenging activity of the HPS was calculated according to the following equation:

(3) $$\mathrm{scavenging}\;\mathrm{percentage}\;(\%)=\left[{(A}_{2}-A_{3})/(A_{1}-A_{3})\right]\times 100\%$$

#### 2.6.3. Determination of Superoxide Anion Radical Scavenging Rate by HPS

The HPS sample was prepared with distilled water into 3, 6, 9, 12, and 15 mg/mL sample solutions with five concentrations. According to the operating instructions of the kit, we added the reagent and prepared the blank tube and the measuring tube. The absorbance was measured at 530 nm after reacting at 37 °C for 20 min after full mixing and denoted as *A*_1_ and *A*_2_. The superoxide anion radical scavenging activity of the HPS was calculated according to the following equation:

(4) $$\mathrm{scavenge}\;\mathrm{percentage}\;(\%)=\left[{(A}_{1}-A_{2}\right)/A_{1}]\times 100\%$$

### 2.7. Statistical Analysis

The software SPSS 27.0 was used for data analysis, and data were expressed as mean ± SD. The drawing tool was GraphPad 8.02. Significant differences between the control and experimental groups were compared by one-way ANOVA followed by the Duncan test. A difference of *p* < 0.05 was considered statistically significant. 

## 3. Results and Discussion

### 3.1. Extraction of HPS

**Figure 2:**
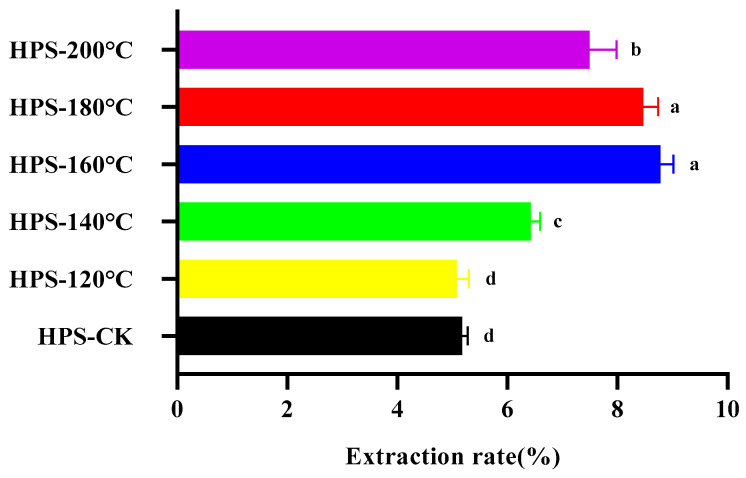
The effect of SE temperature on the extraction rate of HPS. The same letter means that there was no significant change in different concentrations in the same group, and different letters mean that there were significant differences in different concentrations in the same group.

HPS were extracted by water extraction and alcohol precipitation, and the protein, pigment and small molecular impurities were removed. As shown in Figure 2, after SE pretreatment, the yield of HPS changed significantly. With the increase in SE pretreatment temperature, the extraction rate of HPS increased initially and then decreased slightly, reaching a maximum at 160 °C (8.78 ± 0.24%), which was 69.50% higher than that of HPS-CK (5.18 ± 0.10%). Except for HPS-120 °C, the extraction rate of other groups was more elevated than HPS-CK. This is because immediate depressurization destroys the cell walls’ structure, which facilitates the extraction of polysaccharides, consistent with the results of Wheat Germ polysaccharides obtained by SE pretreatment [34]. The reason that the extraction rate decreased slightly after 160 °C may be the high temperature, which results in the degradation of macromolecular polysaccharides, reducing the generation of HPS. Ye et al. only used the traditional hot water extraction method to extract *H. marmoreus* polysaccharides (HMP) and the yield was only 4.8% [35]. Therefore, SE treatment of *H. marmoreus* is a pretreatment method that can effectively improve the yield of polysaccharides.

### 3.2. Analysis Results of HPS Components

#### 3.2.1. Molecular Weight of HPS

**Figure 3:**
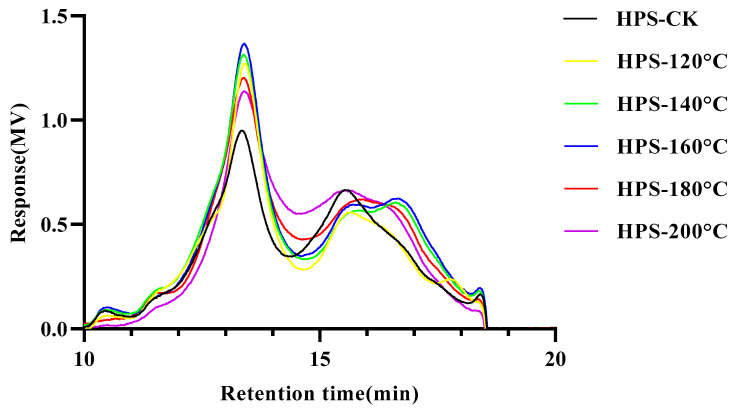
Gel permeation chromatography (GPC) chromatograms of HPS.

The GPC diagram of the HPS is shown in Figure 3 and Table 1. It can be seen from the figure that the HPS present multiple asymmetric peaks. The peak time of HPS was between 13 and 14 min, indicating that an uneven polysaccharide yield was obtained. Interestingly, the HPS extracted from *H. marmoreus* after SE pretreatment showed lower Mw than those from HPS-CK, and Mw decreased with the increase in SE treatment temperature. This is because SE pretreatment destroyed the glycosidic bond of *H. marmoreus* and gradually degraded the high molecular weight polysaccharides into low molecular weight polysaccharides [14]. Consistent with the results of Liu et al. [18], the molecular weight of *Ampelopsis grossedentata* polysaccharides decreased slightly with SE pretreatment, indicating that some high molecular weight polysaccharides were gradually degraded into low molecular weight polysaccharides and oligosaccharides, showing higher biological activity. In addition, SE pretreatment did not affect the MP of the HPS, but Mn and Mz decreased with the rise in SE pretreatment temperature, possibly because of the increase in low molecular weight components and the reduction in the molecular weight distribution range of HPS. When SE pretreatment temperature reaches 180–200 °C, the PD value of HPS-180 °C and HPS-200 °C decreases compared with HPS-CK, possibly due to the extremely narrow molecular weight distribution caused by the high SE pretreatment temperature. In conclusion, SE pretreatment reduced the molecular weight of HPS, suggesting that they have higher biological activity.

#### 3.2.2. Monosaccharide Composition of HPS

The monosaccharide compositions of the HPS are shown in Table 2 and Figure 1. The HPS were mainly composed of Man, Gal, Glc, Fuc, and other monosaccharides. The Glc content of the HPS first decreased, then increased with the increase in SE pretreatment temperature, and reached the minimum when the temperature reached 160 °C. Interestingly, the contents of Man, Gal, and Fuc increased first and then decreased with the increase of SE pretreatment temperature, reaching a peak value at 160 °C. These results suggested that SE pretreatment modifies the percentage of monosaccharide composition in HPS, especially HPS-160 °C. After SE pretreatment at 160 °C, the contents of Man, Gal, and Fuc in HPS were increased, and the contents of Glc in HPS were decreased. Zhang et al. [36] studied the structure and immunomodulatory activity of polysaccharides from *H. marmoreus*, and the results of monosaccharide composition showed that those with lower Glc content had higher biological activity. Xu et al. [37] found that among auriculae polysaccharides, the polysaccharides with the highest Man molar ratio had higher antioxidant activity. This could be because polysaccharides with high content of Man can easily reduce the secretion of nitric oxide (NO) and reactive oxygen species (ROS), thus improving the antioxidant activity of the polysaccharides [38]. In addition, studies have also shown that the higher the content of Gal and Fuc in polysaccharides of *H. marmoreus*, the stronger the antioxidant activity [36]. In conclusion, SE pretreatment increased the content of monosaccharides (e.g., Man, Gal, and Fuc) with antioxidant activity in the HPS, suggesting that SE pretreatment could improve the antioxidant activity of HPS.

#### 3.2.3. Protein and Total Sugar Content of HPS

The SE pretreatment temperature increased, and the total sugar content of the HPS increased (Table 3). The HPS-200 °C was the highest (83.22 ± 2.49%), which was 16.23% °C higher than that of the HPS-CK (71.60 ± 1.06%). The total sugar contents of the HPS-160, HPS-180 °C, and HPS-200 °C were significantly higher than that of the HPS-CK (*p* < 0.05). This might be due to damage to the cell wall structures of the *H. marmoreus* caused by the increase in SE pretreatment temperature, which leads to the enlargement of cell gaps and facilitates the release of polysaccharide components from the large and porous cell walls. As shown in Table 3, SE pretreatment resulted in lower protein content and higher total sugar content of the HPS compared to the HPS-CK. This is consistent with the results of Xi et al.‘s study on the effect of SE pretreatment on the protein and total sugar contents of barley bran dietary fiber, indicated that SE pretreatment can increase the total sugar content of polysaccharides and reduce the protein content [39].

### 3.3. Analysis Results of HPS Structural Characteristics

#### 3.3.1. Infrared Spectroscopy (FT-IR) of HPS

**Figure 4:**
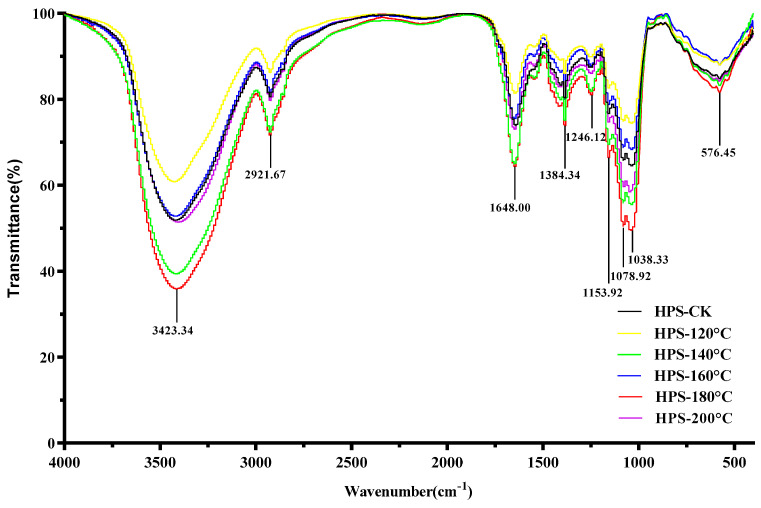
FT-IR spectrum of HPS.

Figure 4 shows the infrared spectroscopy of the HPS. As can be seen from Figure 4, SE pretreatment did not change the spectral distribution of the HPS, but only changed the intensity of the absorption peak, indicating that the primary functional group of polysaccharides did not change after SE pretreatment. The result is congruent with Wang et al.’s [40] findings on the dietary fiber of *Poria cocos* peel residue after SE pretreatment. The HPS had strong absorption peaks at 3423.34, 2921.67, 1648.00, 1384.34, 1246.12, 1153.92, 1078.92, 1038.33, and 576.45 cm^−1^. The strong absorption at 3423.34 cm^−1^ belonged to the tensile vibration of O–H, indicating that HPS have intermolecular hydrogen bond forces, and the stronger the absorption peak, the greater the hydrogen bond force [41]. The peak at 2921.67 cm^−1^ represented the stretching C–H vibration [42]. The strong absorption at 1648.00 cm^−1^ was due to asymmetric tensile vibration in the C=O band [43]. The peak at 1384.34 cm^−1^ was due to the deformation vibration of C–H [44]. C–H is a functional group that is not easily oxidized and C–H bonds have lower bond dissociation energies than O–H bonds, and so they can provide a certain degree of antioxidant activity for polysaccharides [45]. The peaks at 1246.12 cm^−1^ were mainly caused by N=O and N=N tensile vibrations, C–N tensile vibrations, and C–H variable angle vibrations [46]. Three characteristic absorption peaks at 1153.92, 1078.92, and 1038.33 cm^−1^ were the characteristic absorption peaks of polysaccharides, representing the stretching vibration of C–O, C–O–C, or C–O–H, respectively, of the pyranose ring, among which C–O–H is the functional group mainly responsible for the antioxidant activity of polysaccharides [47,48]. This indicated that the HPS had polysaccharide characteristic structures, with the HPS polysaccharide characteristic structures extracted from *H. marmoreus* after SE pretreatment at 160–200 °C being more pronounced. It was worth noting that the absorption peak intensities of C–H and C–O–H in the HPS extracted from *H. marmoreus* by SE pretreatment at 160–200 °C were higher than those of HPS-CK, indicating that SE pretreatment can enhance the antioxidant activity of HPS by increasing the content of antioxidant groups in HPS.

#### 3.3.2. Thermal Analysis of HPS

**Figure 5:**
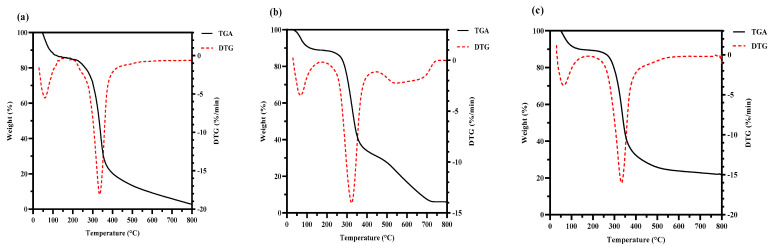

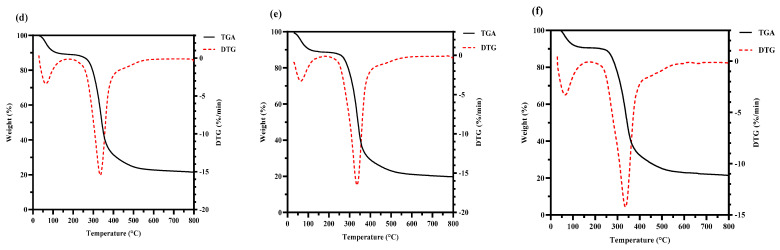
Thermogravimetry analysis (TGA) curves and derivative thermogravimetric analysis (DTG) curves of HPS-CK (**a**); HPS-120 °C (**b**); HPS-140 °C (**c**); HPS-160 °C (**d**); HPS-180 °C (**e**); and HPS-200 °C (**f**).

The thermal stability of the HPS is shown in Figure 5. Compared with HPS-CK, the initial thermal degradation temperature of the HPS extracted from *H. marmoreus* after SE pretreatment was higher (HPS-120 °C, 335 °C; HPS-140 °C, 333.28 °C; HPS-160 °C, 335 °C; HPS-180 °C, 335.65 °C; and HPS-200 °C, 336 °C). In the range of 175–500 °C, weight loss increased because of the thermal decomposition of polysaccharides, and the weight loss rate of HPS-CK was 72.223%. Because of dehydroxylation or deoxidation degradation of the polysaccharide structure, a large amount of weight loss was observed [49]. Compared with HPS-CK, the weight loss of HPS decreased (HPS-120 °C, 61.364%; HPS-140 °C, 63.811%; HPS-160 °C, 64.541%; HPS-180 °C, 65.734%; and HPS-200 °C, 65.425%). These results showed that the HPS extracted from *H. marmoreus* after SE pretreatment had good thermal stability. The possible reason was that some macromolecular substances polymerize or aggregate after SE pretreatment, and these substances have strong heat resistance and are not easily decomposed at 500 °C. The results are consistent with those of the thermal stability of polysaccharides extracted from wheat germ after SE pretreatment, which showed that polysaccharides with higher thermal stability have higher biological value [20].

### 3.4. Antioxidant Activity of HPS In Vitro

#### 3.4.1. DPPH Radical Scavenging Rate of HPS

**Figure 6:**
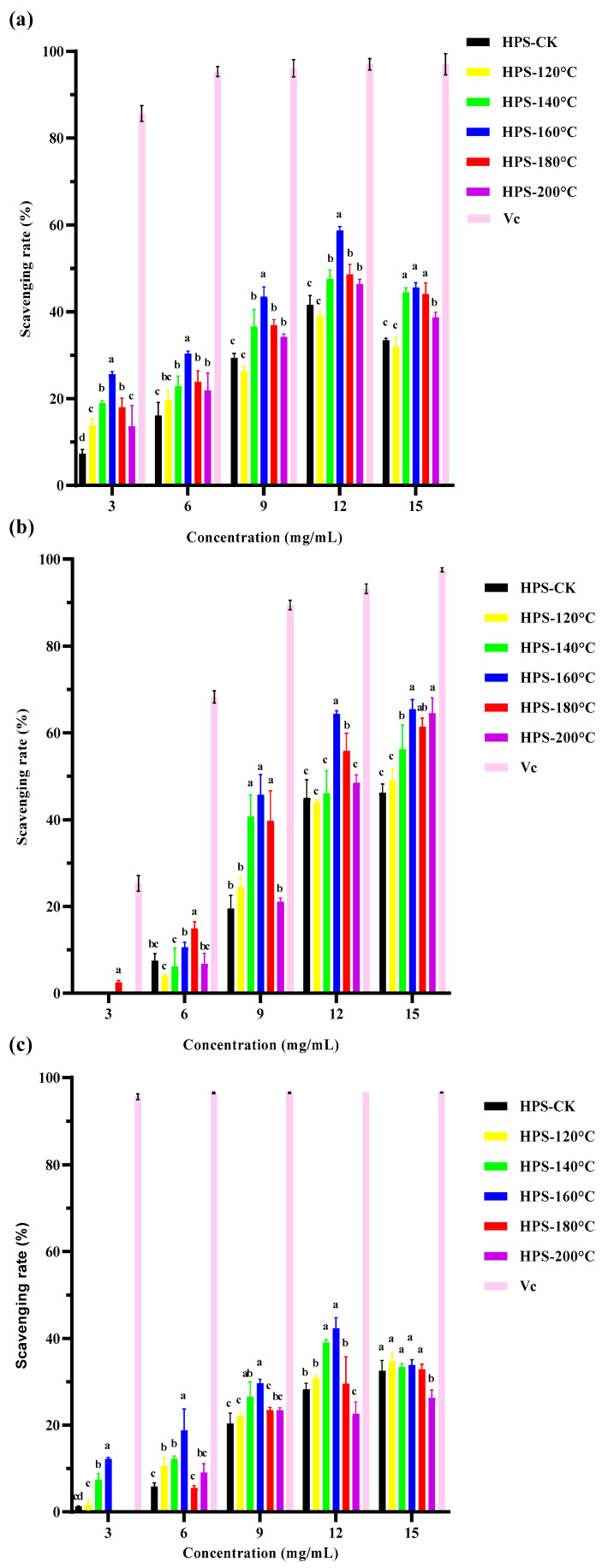
(**a**) DPPH (1,1-diphenyl-2-picrylhydrazyl) radical scavenging capacity of HPS; (**b**) hydroxyl radical scavenging capacity of HPS; (**c**) superoxide anion radical scavenging capacity of HPS. The same letter means that there were no significant changes in different concentrations in the same group, and different letters mean that there were significant differences in different concentrations in the same group.

The principle of polysaccharide removal of DPPH free radicals is that the hydrogen donor reacts with DPPH radicals to form a stable substance that terminates the free radical chain reaction [50]. In high concentrations, polysaccharides can react with DPPH free radicals, effectively remove DPPH free radicals, and exert antioxidant effects. As shown in Figure 6a, the scavenging rate of DPPH free radicals by the HPS increased with increasing concentrations, and HPS extracted from *H. marmoreus* after SE pretreatment were better than HPS-CK at all concentrations (*p* < 0.05). The highest clearance rate was 58.76 ± 0.86% when HPS-160 °C was 12 mg/mL (HPS-CK was 41.54 ± 2.20%). After 12 mg/mL, the clearance rate decreased slightly and gradually stabilized, but the clearance rate was much lower than that of the same concentration of ascorbic acid (97.02 ± 0.96%). These results indicated that HPS-160 °C had a strong scavenging rate of DPPH free radicals. This was consistent with the results for monosaccharide composition. SE pretreatment of *H. marmoreus* at 160 °C increased the contents of Man and Gal in the extracted HPS, decreased the content of Glc, and thus enhanced the scavenging rate of HPS-160 °C on DPPH free radicals. This was because Man and Gal are monosaccharides with high antioxidant activity among polysaccharides [51,52]. In addition, FT-IR results also showed that after SE pretreatment, the content of antioxidant functional groups in the HPS extracted from *H. marmoreus* increased, which may have reduced the hydrogen supply to the HPS, thereby increasing the scavenging rate of DPPH free radicals by the HPS. 

#### 3.4.2. Hydroxyl Radical Scavenging Rate of HPS

Hydroxyl free radicals are the most toxic and harmful free radicals produced in the metabolism of organisms, and can cause serious oxidative damage to proteins, DNA, and lipids in organisms. The removal of hydroxyl free radicals is essential to protect organisms and polysaccharides can cause hydrogen donors or electrons to scavenge hydroxyl radicals [53]. In particular, the polysaccharides extracted after SE pretreatment have a strong ability to clear hydroxyl radicals. Hu et al. [20] also found that SE pretreatment significantly improved the ability of wheat bran polysaccharides to scavenge hydroxyl radicals. It can be seen from Figure 6b, that the HPS extracted by SE pretreatment exhibited concentration dependent enhancement of hydroxyl radical scavenging efficiency. But at the same concentration, the clearance rate of HPS was still lower than ascorbic acid at the same concentration (93.16 ± 0.76%). It is worth noting that when the concentration of HPS-160 °C is 12 mg/mL, the clearance rate of hydroxyl radicals reaches its highest, which is 65.44 ± 1.71%. This could be due to the roughness, looseness, and expansion of the HPS extracted from *H. marmoreus* after SE pretreatment at 160 °C, which were conducive to exposure to binding sites and groups, meaning that monosaccharides such as Man, Gal and Fuc can promote the oxidation of metal ions through electron chelation. Moreover, there was a strong chelation with Fe^2+^, which makes it difficult for Fe^2+^ to be oxidized to Fe^3+^, thus enhancing the antioxidant activity of HPS [8]. After SE pretreatment, the molecular weight of the HPS decreased and the content of Man increased, also leading to an increase in the hydroxyl radical scavenging rate. This is consistent with Ji et al.‘s study on the effect of *Ziziphus Jujuba* polysaccharides on hydroxyl radical scavenging. Ji et al. extracted five different polysaccharide components (GZMP-1, GZMP-2, GZMP-3, GZMP-4, and ZMP) from *Ziziphus Jujuba* and found that the polysaccharides with smaller molecular weight and higher Man content had more obvious scavenging effects on hydroxyl free radicals [54]. Liu et al. only used a hot water extraction method to extract polysaccharides of *H. marmoreus* (PHM) and ascertained that the clearance rate of hydroxyl free radicals of PHM was only 7.6–10.4% [55]. In summary, SE pretreatment can effectively improve the scavenging ability of HPS for hydroxyl free radicals.

#### 3.4.3. Superoxide Anion Radical Scavenging Rate of HPS

Although superoxide anions are weak oxidizing agents, they can decompose to form stronger ROS, such as hydroxyl radicals, causing oxidative stress, cell damage, lipid peroxidation, and other pathological incidents [56]. Therefore, the ability to remove superoxide anions is one of the important indicators of the antioxidant activity of materials. As shown in Figure 6c, the clearance rate of superoxide anions by HPS was concentration dependent. At a concentration of 12 mg/mL, the scavenging rate peaked at HPS-160 °C (42.32 ± 1.73%), which was significantly higher than that at other concentrations and that of HPS-CK (*p* < 0.05), but much lower than for ascorbic acid (96.55%). This was because the polysaccharide structure contains aldehydes or ketones that can release electrophilic groups, releasing C–H and C–O–H stable hydrogen ions, thereby stabilizing superoxide anion radicals [57,58]. The FT-IR results of HPS showed that the C–H and C–O–H contents of HPS extracted after SE pretreatment were higher than those of HPS-CK, indicating that SE pretreatment released C–H and C–O–H stable hydrogen ions in HPS, thereby improving the clearance rate of superoxide anion free radicals by HPS. This is consistent with the results of Ji et al.’s study on laver polysaccharides from different habitats and harvesting periods. They found that although these laver polysaccharides had similar functional groups, those containing more C–H had stronger antioxidant activity [59]. In conclusion, SE pretreatment can significantly improve the scavenging rate of HPS on DPPH free radicals, hydroxyl free radicals and superoxide anion free radicals, suggesting that SE pretreatment can improve the antioxidant activity of HPS in vitro.

## 4. Conclusions

The structural characteristics and in vitro antioxidant activity of HPS extracted from *H. marmoreus* before and after SE pretreatment were studied. In this study, compared with natural polysaccharides, the primary structures (functional groups and monosaccharide composition) of the HPS extracted after SE pretreatment of *H. marmoreus* did not change significantly. However, the percentages of monosaccharide composition changed. We found that the yields and thermal stabilities of HPS extracted from *H. marmoreus* after SE pretreatment were significantly improved. The HPS extracted from SE pretreated *H. marmoreus* showed stronger antioxidant activity than that without HPS-CK, indicating that the structural changes of HPS extracted from SE pretreated *H. marmoreus* tended to enhance antioxidant activity. In conclusion, the pretreatment by SE is an efficient method for extracting active HPS, can significantly improve the yield of HPS and can change its structure to make it have stronger antioxidant activity. However, there are still the following aspects to be further studied in the future: (1) The structures of polysaccharides are complex, and the structures of HPS have only been preliminarily analyzed in this paper. Further separation and purification of HPS are needed in the future, and the linking mode between the monosaccharide residues and the relationship between them and the antioxidant activity of HPS should be studied by means of methylation, GC-MS, and nuclear magnetic resonance. (2) This paper only discussed the antioxidant activity of HPS in vitro, and the antioxidant activity of HPS in vivo can be further studied through animal experiments and other methods.

## Figures and Tables

**Table 1:** Molecular weight of HPS.

Sample	MP/Da	Mn/Da	Mw/Da	Mz/Da	PD
HPS-CK	1.5810 × 10^4^	1.6871 × 10^4^	4.0555 × 10^4^	14.7122 × 10^4^	2.4
HPS-120 °C	1.4782 × 10^4^	1.4557 × 10^4^	3.6219 × 10^4^	13.4053 × 10^4^	2.5
HPS-140 °C	1.5274 × 10^4^	1.3930 × 10^4^	3.4714 × 10^4^	13.1243 × 10^4^	2.5
HPS-160 °C	1.4999 × 10^4^	1.4454 × 10^4^	3.5189 × 10^4^	14.0660 × 10^4^	2.4
HPS-180 °C	1.5219 × 10^4^	1.2196 × 10^4^	2.6802 × 10^4^	8.2788 × 10^4^	2.2
HPS-200 °C	1.5026 × 10^4^	1.2518 × 10^4^	2.2318 × 10^4^	5.3319 × 10^4^	1.8

Note. Mp—the molecular weight of the peak position; Mn—the molecular weight of the number average; Mw—the molecular of the weight average; Mz—the molecular of the z average; PD—the molecular weight distribution.

**Table 2:** The monosaccharide components of HPS.

Sample	Percentage of Monosaccharide Composition (%)							
	Man	Rib	GlcA	Glc	Gal	Xyl	Ara	Fuc
HPS-CK	7.2	1.0	2.4	65.8	17.6	0.1	0.5	5.4
HPS-120 °C	8.5	1.0	2.1	61.5	20.8	0.1	0.5	5.5
HPS-140 °C	8.7	1.4	1.6	60.7	20.6	0.2	0.4	5.6
HPS-160 °C	8.8	1.6	1.6	60.1	21.8	0.2	0.4	6.2
HPS-180 °C	6.0	1.5	2.3	71.3	14.5	0.3	0.3	3.8
HPS-200 °C	6.9	1.5	2.5	71.1	13.7	0.6	0.3	3.3

**Table 3:** The protein and total sugar content of HPS.

Sample	Total Sugar Content	Protein Content
HPS-CK	71.60% ± 1.06% ^d^	4.00% ± 0.57% ^a^
HPS-120 °C	72.32% ± 0.70% ^d^	2.94% ± 0.53% ^ab^
HPS-140 °C	73.89% ± 0.78% ^cd^	3.67% ± 0.90% ^ab^
HPS-160 °C	76.26% ± 2.07% ^bc^	2.81% ± 0.18% ^b^
HPS-180 °C	78.21% ± 1.63% ^b^	3.24% ± 0.66% ^ab^
HPS-200 °C	83.22% ± 2.49% ^a^	2.61% ± 0.16% ^b^

Note. Each value is represented as mean ± SD of triplicate tests (n = 3). The same letter means that there were no significant changes in different concentrations in the same group, and different letters mean that there were significant differences in different groups.

## Data Availability

The original contributions presented in the study are included in the article, further inquiries can be directed to the corresponding author.
